# Comment on Topyła-Putowska et al. Echocardiography in Pulmonary Arterial Hypertension: Comprehensive Evaluation and Technical Considerations. *J. Clin. Med.* 2021, *10*, 3229

**DOI:** 10.3390/jcm11123337

**Published:** 2022-06-10

**Authors:** Giovanna Manzi, Carmine Dario Vizza, Roberto Badagliacca

**Affiliations:** Department of Clinical, Anesthesiological and Cardiovascular Sciences, Sapienza University of Rome, Viale del Policlinico, 155, 00161 Rome, Italy; giovannamanzi91@gmail.com (G.M.); dario.vizza@uniroma1.it (C.D.V.)

**Keywords:** echocardiography, pulmonary arterial hypertension, right ventricle

## Abstract

A comprehensive PAH echocardiographic examination of patients with pulmonary arterial hypertension (PAH) should include a set of parameters resembling the pathophysiological changes occurring in the course of the disease. This approach could help clinicians build a complete picture of the patient, test the effects of targeted therapies and identify patients who need a more aggressive therapeutic approach to achieve a low risk-status.

We have read with great interest the review recently published in the *Journal of Clinical Medicine* by Topyła-Putowska et al. [1], describing the different echocardiographic techniques used to evaluate patients with pulmonary arterial hypertension (PAH). Given its low cost and wide availability, echocardiography is crucial as the first-line modality in the assessment of PAH patients, from diagnosis to prognosis. However, large-scale multicenter studies investigating the role of echocardiography for prognostic stratification of PAH patients are still missing.

The description of PAH pathophysiology is pivotal to understanding what a complete echocardiographic assessment of PAH patients should include [2,3].

In the earlier stages, RV preserves cardiac output, increasing its contractility and developing compensatory hypertrophy, with little or no increase in right-heart-chamber dimensions. In the later stages, the homeometric adaptation fails and the right ventricle (RV)/pulmonary artery (PA) coupling is no longer preserved. RV volumes progressively increase, leading to functional tricuspid regurgitation, under-filling of the left ventricle and finally to heart failure [4].

Thus, PAH echocardiographic assessment should include parameters resembling this pathophysiological behavior in each patient [5,6], checking for changes in the same direction, whether there is improvement or worsening during the course of the disease.

A set of pathophysiologically sound parameters is therefore missing in the echocardiographic evaluation of PAH patients proposed by the Authors.

1. Non-invasive echocardiographic measurements assessing RV systolic function, the main determinant of PAH patients’ prognosis, are largely discussed in the review. However, the authors made no mention to the TAPSE/PASP ratio, a straightforward echocardiographic surrogate of RV–pulmonary artery coupling that was significantly associated with pulmonary hemodynamics, WHO functional class and outcome in PAH [7]. As recently reported, improvement of TAPSE/PASP ratio under PAH therapies is associated with a significant reduction in PVR and the likelihood of achieving a low-risk status [8].
2. Robust evidence on the effect of chronic increased afterload on RV diastolic function is limited in PAH. Nevertheless, Badagliacca et al., examining 108 patients with idiopathic PAH, identified three RV post-systolic strain patterns associated with different clinical presentation, hemodynamics and outcome [9]. The pattern characterized by a slow return of strain–time curves to baseline during diastole identified patients with a more advanced stage of PH with right heart failure and a lower rate of freedom of clinical worsening. Although further investigation is needed to confirm the prognostic role of RV post-systolic strain, its assessment could help physicians in the risk stratification of PAH patients.
3. The delayed contraction of basal and mid-RV free wall results in RV dyssynchrony (RVD) [10], an independent prognostic factor in PAH associated with a worse functional state, a more impaired hemodynamic profile and poor outcome. RVD is mainly determined by pulmonary vascular resistance (PVR) and RV size; thus, it can be reversed by a remarkable PVR reduction [11]. The echocardiographic RVD evaluation could allow clinicians to predict clinical worsening and evaluate the efficacy of PAH therapy.
4. Finally, echocardiography should help clinicians to monitor PAH patients’ treatment response. Approved therapies largely rely on RV afterload reduction to allow right-heart reverse remodeling (RHRR), defined by the combination of decreased right-sided atrial and RV areas and LV eccentricity. According to the pathophysiological model of afterload mismatch, the likelihood of RHRR after institution of targeted therapies is strongly correlated with a reduction in PVR > 50%, a result that can be achieved through aggressive therapeutic strategies, including parenteral prostacyclins [12,13]. RHRR, which emerged as an independent predictor of prognosis and risk status [14], should therefore be included in the routine imaging assessment of PAH patients.

The interesting review by Topyła-Putowska et al., underlying the role of echocardiography in the work-up of PAH patients, should include all parameters describing the pathophysiological changes occurring throughout the course of PAH. A comprehensive echocardiographic examination could help clinicians build a more complete picture of the patient, beyond routine clinical assessment, and identify higher-risk patients needing a more aggressive therapeutic approach.
